# De Novo Assembly of the *Dirofilaria immitis* Genome by Long-Read Nanopore-Based Sequencing Technology on an Adult Worm from a Canine Cardiopulmonary Dirofilariosis Case

**DOI:** 10.3390/ani12111342

**Published:** 2022-05-25

**Authors:** Sónia Gomes-de-Sá, Patrícia Barradas, Luís Queirós-Reis, Isabel M. Matas, Irina Amorim, Luís Cardoso, Antonio Muñoz-Mérida, João R. Mesquita

**Affiliations:** 1 ICBAS—School of Medicine and Biomedical Sciences, Porto University, 4050-313 Porto, Portugal; soniavilargomessa@gmail.com (S.G.-d.-S.); luisqueirosreis@gmail.com (L.Q.-R.); ifamorim@icbas.up.pt (I.A.); 2 Epidemiology Research Unit (EPIUnit), Instituto de Saúde Pública da Universidade do Porto, 4050-600 Porto, Portugal; patricia.barradas@iucs.cespu.pt; 3 Laboratório para a Investigação Integrativa e Translacional em Saúde Populacional (ITR), 4050-600 Porto, Portugal; 4 Department of Sciences, University Institute of Health Sciences (IUCS), CESPU, CRL, 4585-116 Gandra, Portugal; 5 Centro de Investigação em Biodiversidade e Recursos Genéticos (CIBIO/InBIO), Universidade do Porto, Vairão, 4485-661 Porto, Portugal; isa_mmcc@hotmail.com (I.M.M.); amunoz@cibio.up.pt (A.M.-M.); 6 Institute of Molecular Pathology and Immunology of the University of Porto (IPATIMUP), 4220-135 Porto, Portugal; 7 Institute for Research and Innovation in Health (i3S), University of Porto, 4200-135 Porto, Portugal; 8 Department of Veterinary Sciences, School of Agrarian and Veterinary Sciences, University of Trás-os-Montes e Alto Douro (UTAD), 5000-801 Vila Real, Portugal; lcardoso@utad.pt; 9 Animal and Veterinary Research Centre (CECAV), UTAD, 5000-801 Vila Real, Portugal

**Keywords:** *Dirofilaria immitis*, genome, macrocyclic lactone resistance, long-read

## Abstract

**Simple Summary:**

*Dirofilaria immitis* is a zoonotic parasite that infects canids and other vertebrates. We expanded the use of long-read nanopore-based sequencing technology by performing genomic de novo assembly of a *D. immitis* specimen retrieved from a canine cardiopulmonary dirofilariasis case by using the ONT MinION platform. We also identified loci previously characterized as being associated to macrocyclic lactone resistance selection pressure. The identification of a resistant zoonotic parasite alerts for the overuse of macrocyclic lactone in the region.

**Abstract:**

*Dirofilaria immitis* is a zoonotic parasitic nematode that infects domestic and wild canids, among its vertebrate hosts. The genetic analysis of *D. immitis* nowadays transcends the need for genetic taxonomy of nematodes, such as the study of resistance to macrocyclic lactone. We expanded the use of long-read nanopore-based sequencing technology on nematodes by performing genomic de novo assembly of a *D. immitis* specimen retrieved from a canine cardiopulmonary dirofilariasis case using the ONT MinION platform, followed by the study of macrocyclic lactone resistance. The assembled genome of *D. immitis* consists of 110 contigs with an N50 of 3687191. The genome size is 87899012 and contains a total of 9741 proteins; 6 ribosomal RNAs, with three belonging to the small subunit (18S) and three to the large subunit (28S); and 73 tRNAs. Subsequent analysis of six loci previously characterized as being associated to macrocyclic lactone resistance selection pressure showed that four have a genotype associated with either some loss of efficacy or the resistance phenotype. Considering the zoonotic potential of *D. immitis*, the identification of a resistant parasite alerts for the overuse of macrocyclic lactone in the region, which poses a potential risk to both veterinary and human public health.

## 1. Introduction

*Dirofilaria immitis* is a parasitic nematode that infects domestic and wild canids as well as other animals, including humans. The associated disease typically occurs in temperate, tropical, and subtropical areas of the world, with the agent being transmitted by several mosquito species such as those belonging to the *Culex*, *Aedes*, and *Anopheles* genera [1]. These mosquitoes deposit infective stage larvae (L3) at the biting site, which penetrate the host’s skin. The L3 molt into L4 3–12 days post-infection (dpi), later molting into preadult worms 50–70 dpi, which migrate to the pulmonary artery and right ventricle 70–85 dpi, reaching sexual maturity 120 dpi [2]. Females initiate the production of microfilariae (first larval stage) 6–9 months post-infection, with adults living more than 7 years and microfilariae living up to 2 years [2].

*Dirofilaria immitis* worms can cause canine cardiopulmonary dirofilariasis (also known as heartworm disease in dogs), a widespread disease that can have a fatal outcome if animals are not treated [3]. Moreover, *D. immitis* in dogs represents a risk for the human population, who may suffer from pulmonary dirofilariasis and, in many cases, pulmonary nodules that can be misdiagnosed as malignant tumors [4].

Morphological analysis is commonly used for the differentiation of nematode species, but not without its drawbacks such as scarce distinguishable characters, a circumstance that may hamper their classification to the species or even genus level [5]. This is not necessarily the case for *D. immitis*, which can easily be distinguished from other filarioids, taking into account the cardiac location of adults. Nonetheless, genetic analysis nowadays transcends the need for genetic taxonomy of nematodes. An example is the requirement for ascertaining resistance to macrocyclic lactones, known to have a genetic origin [6]. Macrocyclic lactones such as milbemycin oxime, ivermectin, moxidectin, and selamectin are widely available drugs that are used to prevent the establishment of the L3–L4 *D. immitis* stages in dogs and cats. However, loss of efficacy has been described since 2005, and whole-genome analysis has been performed on *D. immitis* isolates to characterize their genetic profile and differences that could potentially be associated with evident loss of efficacy and resistance [6].

Nematode characterization based on markers such as the internal transcribed spacer (ITS) regions of the ribosomal RNA locus, the 28S large subunit ribosomal RNA gene (28S LSU rRNA), the 18S small subunit ribosomal RNA gene (18S SSU rRNA), and the cytochrome oxidase I gene (coi) followed by Sanger sequencing is a widespread, low-cost approach to ascertain genetic profiles [5]. However, the data generated are limited, and novel sequencing strategies such as single-molecule real-time sequencing or third-generation sequencing have reduced the expenses and the necessary hardware for obtaining thorough data on highly contiguous genome assemblies, permitting comprehensive, near-real-time biomonitoring of samples [7,8].

The aim of the present work was to expand the use of long-read nanopore-based sequencing technology on nematodes by performing genomic de novo assembly of a *D. immitis* specimen retrieved from a canine cardiopulmonary dirofilariasis case using the ONT MinION platform.

## 2. Materials and Methods

### 2.1. Extraction of Parasite DNA

Adult *D. immitis* worms (n = 32) were observed at routine parasitological investigation in the right heart and pulmonary artery of a dog that died from an unrelated cause in the municipality of Caminha, northern Portugal, November 2020. The city of Caminha is located by the mouth of the Minho River (circa 340 km in length), under a wet Atlantic climate, being the area of the country with the highest precipitation, reaching around 1800 mm on average and peaking at more than 3500 mm [9]. The dog hosting the *D. immitis* worms was kept in a municipal kennel since birth and had not received any macrocyclic lactone treatment. No ethics permission was obtained since the parasites were taken during regular post-mortem evaluation. One nematode was selected, washed three times in phosphate-buffered saline at pH 7.2, frozen at −80 °C, and subjected to mechanical disruption with a disposable pestle. Nucleic acid extraction followed the previously described procedures [7]. Briefly, the homogenate was incubated with 20 μL proteinase K (Qiagen, Hilden, Germany) and 180 μL of Buffer ATL (Qiagen) for 48 h at 56 °C, with vortexing (200 rpm) in a thermoblock (Eppendorf Epp Thermomixer; Hamburg, Germany). High-molecular-weight (100–200 kb) DNA was then extracted using a magnetic-bead-based protocol (MagAttract HMW DNA kit; Qiagen) as described by the manufacturer. Eluted DNA (on 100 μL of 10 mM tris-HCL) was evaluated for size distribution on an agarose (0.8%) gel. DNA was assessed on a Nanodrop spectrophotometer (ThermoFisher, Waltham, MA, USA). Genomic DNA size selection was then performed using a 0.4× volume of AmpureXP beads (Beckman Coulter, Brea, CA, USA) in order to remove smaller fragments.

### 2.2. Library Preparation and Sequencing

The 1D genomic ligation (SQK-LSK109) library preparation kit (ONT, Oxford, UK) was used imputing 1.2 μg of extracted genomic DNA, and libraries were then developed as instructed by the manufacturer with a calculated final library quantity assessment at 467 ng. Then, 79.3 ng was loaded onto the MinION sequencer using an R9.4.1 flow cell managed by the MinKNOW software (version 18.12.9, ONT). The ONT MinION Mk1B platform was used with active channel selection performed at every 1.5 h, resourcing to no script modifications. Refueling of the flow cell was performed 24 h after initiation by first extracting excess liquid from the waste chamber, followed by the addition of 37.5 μL of SQB and 37.5 μL of H2O on the SpotON sample port. An additional 24-hour run was then performed.

### 2.3. Base-Calling, Genome Assembly, and Read Alignments

After completion of the sequencing run, Guppy (version 2.3.5, ONT) was used for base-calling signal data (.fast5 files), with the generated fastq files used to generate statistics with NanoPlot (version 1.19.0). Raw reads were processed using Porechop software (version 0.2.4) with the default parameters to trim sequences from all known Oxford Nanopore adapters. After trimming, all reads were used to perform a complete genome assembly using Flye software (version 2.8.3) with the parameters “-nano-raw” to specify the input type data and an expected genome size of 100 Mb. Contigs obtained at the assembly step were processed using Kraken2 to locate potential contaminants in the sample. Some contigs were identified as belonging to *Canis lupus*, *Homo sapiens*, or *Wolbachia* and therefore removed from the dataset for further analyses. Contigs potentially belonging to *D. immitis* were corrected by mapping the raw reads against the assembled contigs through the Pilon software (version 1.24). Confirmation of the species was double-checked by using the small subunit of the ribosomal RNA (18S) against the NR database using NCBI blast (E-value of 0.0 and 98.91% identity with accession AB973231.1) and blast against the SILVA SSU database, where the best match was again AB973231.1 belonging to *D. immitis*. The corrected contigs were compared against the three available *D. immitis* genomes at NCBI (GCA_009829315.1, GCA_001077395.1, and GCA_013365355.1) to verify the integrity of our assembly. The completeness of the assembly was verified through BUSCO software (version 4) using the nematode database. Final contigs were annotated in order to locate coding regions using Genemark-ES software (version 4.65) with self-training and default parameters. Non-coding regions belonging to rRNAs and tRNAs were also located with RNAmmer software (version 1.2) and tRNAscan-SE (version 2.0), respectively. Functional annotation for the coding regions was performed using Sma3s (version 2), retrieving GO terms and EC numbers for enzymes. Deposition of the Whole Genome Shotgun project was performed at DDBJ/ENA/GenBank under the accession code JAKNDB000000000, with the current version (described in the present paper) being JAKNDB010000000.

Single-nucleotide polymorphism (SNP) loci genotypes that differentiate loss of efficacy/resistant populations from susceptible *D. immitis* populations, previously referred to as markers for macrocyclic lactone resistance in *D. immitis* in the United States [6] and in Australia [10], were identified in the *D. immitis* genome with the Basic Local Alignment Search Tool (BLAST) and Interactive Genome Viewer (IGV) for full characterization of the sample.

## 3. Results and Discussion

The assembled genome of *D. immitis* consists of 110 contigs with an N50 of 3687191. The majority of the contigs had a very good match with one or more of the compared genomes without any sign of fragmentation. The genome size is 87899012 and contains a total of 9741 proteins; 6 ribosomal RNAs, with three belonging to the small subunit (18S) and three to the large subunit (28S); and 73 tRNAs. The results obtained for BUSCO show that the genome is complete to a high level, showing the following values (C:93.9% [S:93.5%, D:0.4%], F:1.7%, M:4.4%, n:3131). We therefore present evidence that a single ONT MinION flow cell can produce sufficient data to assemble a contiguous, high-level, near-full-length genome of *D. immitis*. Noteworthily, parasite genome assemblies used as references are still today considered to be vastly fragmented, frequently hampering more in-depth analysis [11].

A total of 42 loci in the *D. immitis* genome, with the corresponding SNP associated with resistance selection pressure or at least loss of efficacy for various macrocyclic lactones [6,10], were studied. Subsequent analysis of the loci associated with macrocyclic lactone resistance selection pressure showed that 27 of the 42 loci have a genotype associated with either some loss of efficacy or the resistance phenotype (Table S1).

Interestingly, the dog hosting the *D. immitis* worm collected for this study was kept in a municipal kennel since birth and had not received any macrocyclic lactone treatment, a fact which suggests that resistance-associated SNPs were likely acquired from ancestral *D. immitis* previously circulating in the region. It is assumed that macrocyclic lactone resistance first appeared circa 1998. At that time, macrocyclic lactone *D. immitis* preventives had been recommended for use as the first line of treatment to prevent heartworm for over 10 years, having been registered as 100% effective [12,13]. Hence, it is generally assumed that the basis for genetic changes causing macrocyclic lactone resistance only arose and was selected in that decade. As such, resistant lines of *D. immitis* are suggested to have been circulating prior the use of macrocyclic lactone preventives [12,13].

True drug resistance has a genetic basis; hence, continuing efforts to analyze *D. immitis* whole genomes from resistant and susceptible lines could help detect genetic markers for resistance [6]. Long-read sequencing technologies allow for democratizing access to powerful sequencing options, reducing the cost of de novo genome assemblies of understudied organisms. The MinION platform was first made available in 2014, presenting a compact, novel, and lightweight sequencing platform that could produce long reads used for real-time base-calling [14]. The sequencer uses a nanopore that holds a biological membrane where DNA is driven while producing differences in electrical current that are measured and translated as different DNA bases [14,15,16]. The ONT MinION platform can not only provide a low-cost option for projects that were previously considered cost-prohibitive but also grants access to this technology in underdeveloped areas that do not have the infrastructural capacity for sequencing. Moreover, the sequence quality seems to allow for the profiling of drug resistance patterns, which allows for more informative treatment options [14].

## 4. Conclusions

In conclusion, this work provides, for the first time, the de novo assembly of the *D. immitis* genome using long-read nanopore-based sequencing technology. We show that a single ONT MinION flow cell can produce sufficient data to assemble a contiguous, high-quality genome from a complex nematode. The data from this study also provide information suggesting the circulation of macrocyclic-lactone-resistant *D. immitis* in northern Portugal. Considering the zoonotic potential of *D. immitis*, the identification of a resistant parasite alerts for the overuse of macrocyclic lactone in the region, which poses a potential risk to both veterinary and human public health.

## Data Availability

All data is presented in the article.

## Supplementary Materials

The following are available online at https://www.mdpi.com/article/10.3390/ani12111342/s1, Table S1. Macrocyclic lactone resistance profile in a Dirofilaria immitis collected from a dog in northern Portugal, November 2020.
